# Implementation Quality of a Positive Youth Development Program: Cross-Case Analyses Based on Seven Cases in Hong Kong

**DOI:** 10.1100/tsw.2008.130

**Published:** 2008-10-10

**Authors:** Daniel T. L. Shek, Rachel C. F. Sun

**Affiliations:** ^1^ Quality of Life Centre, Hong Kong Institute of Asia-Pacific Studies, The Chinese University of Hong Kong, Hong Kong; ^2^ Kiang Wu Nursing College of Macau, Macau; ^3^ Social Welfare Practice and Research Centre, The Chinese University of Hong Kong, Hong Kong

**Keywords:** adolescence, positive youth development, implementation quality, cross-case analyses, Project P.A.T.H.S.

## Abstract

Cross-case analyses of factors that influence the process and implementation quality of the Tier 1 Program of the Project P.A.T.H.S. based on seven cases were carried out. Systematic and integrative analyses revealed several conclusions. First, several factors related to policy, people, program, process, and place (5 “P”s) were conducive to the successful implementation of the Tier 1 Program in the schools. Second, there were obstacles and difficulties with reference to the 5 “P”s that impeded the quality of implementation. Third, policy support and people (especially commitment and passion of the principals, senior school administrators, and program implementers) are two main groups of factors that influence the quality of program implementation. Fourth, although there were different arrangements for program implementation, incorporation of the Tier 1 Program into the formal curriculum was a sound and viable strategy. Fifth, implementation of the Tier 1 Program in schools that admitted students with high or low academic achievement was viable. Sixth, the program was generally perceived positively by the program participants and implementers. Finally, the program implementers perceived the program to be beneficial to the program participants.

What are the factors that affect the success or failure of positive youth development programs? Nation et al.[1] pointed out that there are many factors that determine the success of an adolescent prevention program. Among these factors, process variables, such as varied teaching methods (i.e., use of a wide range of teaching methods that help the program participants to become aware and understand problem behaviors and acquire the related skills) and positive relationships with adults (e.g., workers), are important factors to be considered. There are also research findings that show that teaching practices and program implementation attributes influence the extent of program success. Harachi et al.[2] reported findings to support some of the propositions of the social development model that instructional strategies

(proactive classroom management, cooperative learning methods, strategies to enhance student motivation, student involvement and participation, reading strategies, and interpersonal and problem-solving skills training) were related to student social competencies. Similarly, Tobler et al.[3] investigated what types of program were most effective in reducing, delaying, or preventing marijuana use, and examined whether characteristics of the participants and program implementation factors were related to program success. Results showed that programs with high peer interaction were more effective than programs with low peer interaction and that the delivery method instead of the program content determined the success of the program.

In their discussion of principles of effective family-focused intervention, Kumpfer and Alvarado[4] identified several features of the program, including comprehensive multicomponent interventions, holistic coverage, high dosage, developmentally appropriate, early intervention, incorporation of cultural traditions into the program, which are intrinsic to program success. Furthermore, characteristics of the workers (such as personal efficacy; confidence; affective characteristics of genuineness, warmth, humor, and empathy; ability to structure sessions) and the use of interactive skills training methods (e.g., role plays, active modeling) are important. Finally, empowering clients to identify their own solutions is conducive to supportive relationships and reduces parental resistance and dropout. In their discussion of community interventions and effective prevention, Wandersman and Florin[5] pointed out that the following questions should be asked: Are capacities needed to put this intervention into place with quality? How will the intervention be carried out? How will the quality of implementation be assessed? How well will the intervention work? How shall continuous quality improvement strategies be incorporated? If the intervention is successful, how will the intervention be sustained? Obviously, an in-depth understanding of the factors that influence the quality of program implementation is important.

An integration of the literature shows that there are generally five groups of factors that would affect the outcomes of positive youth development programs. The first group of factors in the 5 "P"s (policy, people, program, process, and place) is related to school **policies**. Obviously, if the school policies are supportive of the program to be implemented, administrative structuring and staff deployment could be more legitimately and efficiently done. On the other hand, if the school adopts a lukewarm or discouraging policy, it would be a detrimental blow to staff morale. On a macro level, if there is external Government policy support for the implementation of positive youth development programs, it would facilitate the implementation of the related programs.

The second group of factors is related to **people**. There are at least four groups of people that would affect the program outcomes. First, school principals and senior administrators are important sources of influence as they shape the policies and administrative structure and support given to the related programs. Second, the program implementers are crucial to program success. The personal beliefs of program implementers obviously determine their motivation to commit. The skills possessed by colleagues who implement the program are also very important. For example, the readiness of teachers or social workers who implement the program in an interactive manner is critical in affecting the classroom atmosphere. Third, other stakeholders, such as parents, are important people who would influence program success. For example, if the parents believe that positive youth development programs are not beneficial to adolescent development, they will not allow their children to join such programs. Finally, the program participants also affect the program success. If the program participants do not think the program is helpful (i.e., psychological rejection) and do not have motivation to participate in the program (i.e., behavioral rejection), they will not benefit from the program.

The third group of factors is related to the **program**. Obviously, if the program is poorly designed, it will not be successful even though there are good policies and people. In contrast, if the development of the program is governed by well-articulated programs and the best available evidence, it will trigger the intended psychosocial mechanisms that will result in positive changes in the program participants. Furthermore, programs that are more likely to bring positive outcomes are those that are more interactive in nature and developmentally appropriate, which can arouse the motivation of the program participants. In cross-cultural contexts, cultural sensitivity and use of appropriate indigenous cultural material are also important.

The fourth group of factors affecting program outcome is related to the implementation **process**. Obviously, even if we can have a supportive policy, committed people, and a program that is guided by good theoretical mechanisms and the best available evidence, the program still may not work if it is implemented poorly. In particular, a program may fail simply because program adherence is not high. Moreover, if the delivery is not regarded as interesting and interactive, the program participants may not be motivated to participate in the program. In addition, other classroom characteristics, such as student discipline, classroom climate, and morale of the students, also make a difference.

Finally, factors related to **place** are important. The implementation of the program in a safe and comfortable environment is crucial, particularly when students are divided into small groups for discussion, role play, and other group activities. As audio-visual materials are commonly used in positive youth development programs, the availability of equipment such as computers and video projectors, and the ease of using them, are important considerations.

In short, factors related to the 5 "P"s influence the outcomes of adolescent prevention and positive youth development programs. Utilizing the conceptual framework based on the 5 "P"s, findings emerging from cross-case analyses based on seven cases reported in this special issue are presented. Besides examining factors that affect the implementation of positive youth development programs, there are two other issues addressed in this paper. First, the question of whether the program can be efficiently and successfully incorporated into the formal curriculum is examined. Second, the question of whether the program can be successfully implemented in schools that admit students with different academic achievement is explored.

## METHODS

### Participants

Among 207 schools that joined the Project P.A.T.H.S. (Secondary 1 Curriculum) in the Full Implementation Phase, seven schools were invited to participate in the case study. All the schools were coeducational schools located in Kowloon, Hong Kong Islands, and the New Territories, and their students were categorized into having low, medium, or high academic achievement. The background characteristics of these seven cases are presented in Table 1.

Although these schools shared some similarities, there were unique features in each case. In the first case (School A) reported by Shek and Sun, the 20-h Tier 1 Program was covered in the class teacher's periods, Life Education subject, and project-based learning. In the second case (School B) reported by Shek, Chak, and Chan, the Tier 1 Program (20 h) was wholly incorporated into the Life Education subject of the formal curriculum. In the third case (School C), Lee reported a case study based on a school in which the 20-h Tier 1 Program was incorporated into Liberal Studies lessons in relation to education policy changes and education reform in Hong Kong.

In the fourth case (School D) conducted by Lam, data were collected from a school that admitted students with high academic achievement. The Tier 1 Program (20 h) was wholly incorporated into the Civic and Moral Education subject of the formal curriculum. In the fifth case (School E) conducted by Lam, data were collected from a school that admitted students with low academic achievement. The 20-h Tier 1 Program was covered in the class teacher's periods, Life Education subject, and activity day. In the sixth case study (School F), Lee reported how the implementers of a school made use of the experience gained in the Experimental Implementation Phase to improve the program implementation quality in the Full Implementation Phase. The 20-h Tier 1 Program was covered in the class teacher's periods, Life Education subject, weekly assembly, and service day. In the final case (School G) reported by Sun, Shek, and Siu, macro school and classroom attributes conducive to program success were examined. In this case, the Tier 1 Program (10 h) was wholly incorporated into the Moral Education subject of the formal curriculum.

**TABLE 1**
**Summary on the Characteristics of Schools that Joined the Case Study**

| School | A | B | C | D | E | F | G |
|---|---|---|---|---|---|---|---|
| | | | | ***Background Characteristics of the School*** | | | |
| **Location (district)** | North Point | Tokuawan | Shatin | Kowloon Tong | Sheung Shui | Tuen Mun | Tuen Mun |
| **Sex composition** | Coed | Coed | Coed | Coed | Coed | Coed | Coed |
| **No. of S1 students (class)** | 119 (three classes) | 190 (five classes) | 215 (five classes) | 200 (five classes) | 200 (five classes) | 200 (five classes) | 120 (four classes) |
| **Academic achievement** | Low | Medium | High | High | Low | Medium | Low |
| | | | | ***Basic Information of Tier 1 Program*** | | | |
| **Choice of program** | 20 h | 20 h | 20 h | 20 h | 20 h | 20 h | 10 h |
| **Mode** | 20 sessions (1 h per session) | 40 sessions (30 min per session) | 20 sessions (1 h per session) | 20 sessions (1 h per session) | Two sessions (2.5 h per session), one session (2 h), and 13 sessions (1 h per session) | 20 sessions (1 h per session) | 20 sessions (30 min per session) |
| **Integration with school curriculum** | Class teacher's period, Life Education, project-based learning | Life Education | Liberal Studies | Civic and Moral Education | Class teacher's period, Life Education, activity day | Class teacher's period, Life Education, weekly assembly, service day | Moral Education |
| **Teacher/social worker as Instructor(s)** | Eight teachers, one teaching assistant, one social worker | Four teachers, one teaching assistant | Three teachers, eight teaching assistants | Five teachers, one social worker | Six teachers, one social worker | 12 Teachers, two social workers | One vice-principal, one teaching assistant, one social worker |
| **Involvement of principal/senior administrative personnel(s)** | Played a supportive role in program implementation | Played a supportive role in program implementation | Played a supportive role in program implementation | Played a supportive role in program implementation | Initiated program implementation | Initiated program implementation and cultivated teachers' involvement | Involved in planning and program delivery |
| **Joined Experimental Implementation Phase** | Yes | Yes | Yes | No | No | Yes | Yes |

## Procedures

The invited schools were required to complete a school background information questionnaire, with both closed- and open-ended questions, to provide basic information about the school, the Tier 1 Program operated in the school, instructors conducting the Tier 1 Program, and implementation details of the Tier 1 Program. The program implementers and school principal of each school were also invited to participate in focus group interviews and individual interviews, respectively, when the school had completed the Tier 1 Program. At the beginning of the interviews, informed consent was obtained from the respondents, the purpose of the case study was explained, and the principle of confidentiality was repeatedly emphasized. Self-constructed semi-structured interview guides for the individual and focus group interviews were designed to collect the data. There are several parts in each interview guide:

- Respondents' perceptions of the school administrative arrangement, such as preparation, support within the school and from the social work agency, and program evaluation
- Respondents' perceptions of the program implementation process
- Respondents' perceptions of program effectiveness, program success, and overall impression

## RESULTS

Systematic and integrative cross-case analyses based on the seven cases reported in this special issue were carried out. Based on the analyses, several observations can be highlighted from the findings. First, the findings based on different cases consistently showed that several factors related to the 5 "P"s were conducive to successful implementation of the Tier 1 Program in the schools. The findings are systematically outlined in Table 2. Second, the findings similarly showed that several factors related to the 5 "P"s impeded the implementation quality of the Tier 1 Program in the schools (Table 3). Third, policy support and people (especially commitment and passion of the principals, senior school administrators, and program implementers) were two main groups of factors that influenced the quality of program implementation. As shown in Table 4, passion and dedication of the program implementers, and support and initiative of the senior administrators (e.g., principals and vice-principals), were decisive factors that influenced program success.

Fourth, the findings showed that all schools incorporated the Tier 1 Program (either 20- or 10-h program) wholly or partially into the formal curriculum. Generally speaking, the findings showed that incorporation of the Tier 1 Program into the formal curriculum was a viable strategy that could generate program success. In Table 5, the advantages and disadvantages of incorporating the Tier 1 Program into the formal curriculum are described. Overall, the informants identified many advantages of incorporating the program into the formal curriculum. Although some disadvantages were identified, they were comparatively minor.

Fifth, regarding the question of whether the program is appropriate for students with different academic achievement, cross-case analyses showed that the program could work in schools that admit students with different levels of academic achievement (Table 6). In schools that admit students with high academic achievement, informants in both schools revealed that the program had positive impacts on students and enhanced teacher-student relationship. In schools that admit students with medium academic achievement, results showed that although students had different learning motivation and their participation varied with topics, they had positive changes after the program. Finally, positive changes were evident in students studying in schools that admit students with low academic achievement.

The sixth observation is that both the program implementers and students generally had positive perceptions of the program. In particular, the students and instructors perceived that the program was beneficial to the program participants (see Table 7). Finally, although some teachers were not certain about the effectiveness of the program, the majority viewed that the program was beneficial to program participants as well as program implementers (Table 8).

## DISCUSSION

Although many positive youth development programs have been developed in the Western context, there are still many unanswered questions concerning factors that influence the implementation quality of such programs[5]. In the present cross-case analyses, a conceptual framework based on the 5 "P"s was used to understand the factors that promoted or impeded program implementation. Consistent with the findings in the literature, the present cross-case analyses revealed that factors with reference to the 5 "P"s influence the quality and success of implementation of positive youth development programs.

Even if we can design positive youth development programs with excellent quality, the outcome of such programs will not be positive if the program is implemented in a poor manner. According to Shek et al.[6], several questions should be considered when the issue of program implementation quality is under consideration: What are the process variables (such as principal support and interdisciplinary collaboration) that influence the quality of the implementation? To what extent is the program implemented according to the original design? In other words, what is the degree of program adherence and program fidelity? Is the morale of the program implementers high? If not, how can we promote the morale of the program implementers? Obviously, the present integrative analyses provide some constructive answers to these questions.

**TABLE 2**
**Factors Promoting the Quality of Program Implementation**

| | | School | | | | | | |
|---|---|---|---|---|---|---|---|---|
| **Aspect** | **Factors Facilitating the Implementation Process** | **A** | **B** | **C** | **D** | **E** | **F** | **G** |
| (i) **Program** | ● Comprehensive program | ✓ | ✓ | | ✓ | | ✓ | ✓ |
| | ● Activities matching students' developmental needs | ✓ | | ✓ | ✓ | | ✓ | ✓ |
| | ● Perceived helpfulness | ✓ | ✓ | ✓ | ✓ | ✓ | ✓ | ✓ |
| | ● Diverse activities | ✓ | ✓ | ✓ | ✓ | ✓ | ✓ | ✓ |
| | ● Ready-made teaching manuals | ✓ | ✓ | ✓ | | | | ✓ |
| | ● Attractive and varied teaching materials | | ✓ | ✓ | ✓ | ✓ | | |
| | ● Developed tailor-made school-based curriculum | | ✓ | ✓ | ✓ | | | |
| | ● Practical and useful program | | | ✓ | | | | |
| (ii) **People** | ● Support for the program and program rationales | ✓ | ✓ | ✓ | ✓ | ✓ | ✓ | ✓ |
| | ● Student-centered, responsible | ✓ | | ✓ | ✓ | ✓ | | ✓ |
| | ● Passionate and motivated to implement the program | ✓ | ✓ | ✓ | ✓ | ✓ | ✓ | ✓ |
| | ● Prior experiences in running similar programs | ✓ | ✓ | | | ✓ | ✓ | ✓ |
| | ● Trust and cooperation among instructors | ✓ | ✓ | ✓ | ✓ | ✓ | ✓ | ✓ |
| | ● Supportive school principal | ✓ | ✓ | ✓ | ✓ | ✓ | ✓ | ✓ |
| | ● Responsible coordinator who has leadership skills | ✓ | ✓ | ✓ | ✓ | ✓ | ✓ | ✓ |
| | ● Students with motivation to learn | ✓ | ✓ | ✓ | ✓ | | ✓ | ✓ |
| | ● Good teamwork | | ✓ | ✓ | ✓ | | | |
| | ● Dedicated teachers | | ✓ | | ✓ | ✓ | ✓ | ✓ |
| | ● Trained teachers | | | ✓ | ✓ | ✓ | ✓ | ✓ |
| | ● Have a sense of ownership | | | | | ✓ | ✓ | |
| | ● Have positive beliefs about students | | | | | ✓ | | ✓ |
| (iii) **Process** | ● Collaboration between school and social work agency | ✓ | ✓ | ✓ | ✓ | | ✓ | ✓ |
| | ● Adequate support from social work agency | ✓ | | ✓ | ✓ | ✓ | ✓ | ✓ |
| | ● Coteaching | ✓ | ✓ | ✓ | ✓ | | ✓ | ✓ |
| | ● Good preparation before teaching | ✓ | ✓ | ✓ | ✓ | ✓ | ✓ | ✓ |
| | ● Varied teaching techniques | ✓ | ✓ | ✓ | | | ✓ | ✓ |
| | ● Worksheets handed in and kept in students folders | ✓ | ✓ | | | | ✓ | ✓ |
| | ● Check students' assignments and give feedback | ✓ | ✓ | | | | ✓ | ✓ |
| | ● Good communication | ✓ | ✓ | ✓ | ✓ | | ✓ | |
| | ● Good debriefing | | ✓ | | | | | |
| | ● Continuous assessment of students' learning | | ✓ | | | | | |
| | ● Good support from teaching assistant | | ✓ | ✓ | | | | ✓ |
| | ● Good teacher-student relationship | | | ✓ | | ✓ | | ✓ |
| | ● Good time management | | | ✓ | | | | |
| | ● Briefing provided for untrained teachers | | | | | | ✓ | |
| | ● Continuous quality improvement | | | | | | ✓ | |

*Table 2 continues*

**TABLE 2 (continued)**

| | | | | | | | | |
|---|---|---|---|---|---|---|---|---|
| (iv) **Policy** | • Reasonable program arrangement | ✓ | ✓ | ✓ | ✓ | ✓ | ✓ | ✓ |
| | • Effective manpower deployment | ✓ | ✓ | ✓ | ✓ | ✓ | ✓ | ✓ |
| | • Specialist teaching | ✓ | | | | | ✓ | |
| | • Structured teams with person-in-charge | ✓ | ✓ | ✓ | ✓ | ✓ | ✓ | |
| | • Meetings and evaluation on program implementation | ✓ | | ✓ | ✓ | ✓ | ✓ | ✓ |
| | • Documentation of administrative procedure | ✓ | | | | | | |
| | • Collaborative decision making | ✓ | | ✓ | ✓ | ✓ | ✓ | |
| | • Buy-in from the teachers | ✓ | ✓ | | ✓ | | | ✓ |
| | • Incorporated the program into formal curriculum | ✓ | ✓ | ✓ | ✓ | ✓ | ✓ | ✓ |
| | • Cooperation among subjects within school | | | ✓ | | | ✓ | |
| | • Teacher support measures | | | ✓ | | | | |
| | • Prior planning | | | | ✓ | ✓ | | ✓ |
| | • Streamlined administration | | | | | | ✓ | |
| (v) **Place** | • Sufficient hardware equipment for running activities | ✓ | | ✓ | | | ✓ | ✓ |
| | • Physical proximity for teachers' communication | ✓ | | | | | ✓ | |
| | • Dedicated school culture | ✓ | ✓ | ✓ | ✓ | ✓ | ✓ | ✓ |
| | • Good support within school | | ✓ | ✓ | ✓ | ✓ | ✓ | |
| | • Appealing classroom environment | | | | | | | ✓ |
| | • Sufficient space and room for running activities | | | ✓ | | | ✓ | |
| | • Provision of facilities to promote students' learning | | | | | ✓ | | |

There are three contributions of this study involving cross-case analyses. First, it provides support for the consolidated conceptual model and it enhances our understanding of the factors that influence the implementation of positive youth development programs. Second, this is the first scientific study of factors intrinsic to the 5 "P"s model in the Chinese context. Third, consistent with the previous findings based on subjective outcome evaluation[7,8], qualitative evaluation[9], process evaluation[10], and interim evaluation[11], the present findings of the cross-case analyses provide additional support for the value and positive impacts of the program.

Nevertheless, the limitations of this integrative case study should be realized. First, the findings of this study are limited to seven cases only. As such, the generalizability of the findings should be viewed with caution. Second, the findings were based on the responses of the principals, vice-principals, teachers, and social workers. If resource permits, similar case studies based on the students should be attempted. Finally, the present case study was based on data collected from one time point. It would be exciting if data could be collected in a longitudinal manner in future. Despite these limitations, the present pioneering study on the factors that influence the implementation of a positive youth development program is an important addition in the Chinese literature.

## ACKNOWLEDGMENTS

The preparation for this paper and Project P.A.T.H.S. were financially supported by The Hong Kong Jockey Club Charities Trust.

**TABLE 3**
**Obstacles Impeding the Quality of Program Implementation**

| Aspect | Obstacles Affecting the Quality of Implementation | School | | | | | | |
|---|---|---|---|---|---|---|---|---|
| | | A | B | C | D | E | F | G |
| (i) **Program** | • Perceived overload of activities | ✓ | | | | | | |
| | • Perceived doubtful linkage between some topics | ✓ | ✓ | | | | | |
| | • Perceived inadequate variety in the activity format | ✓ | | | | | | |
| | • Perceived inflexibility | ✓ | | | | | | |
| | • Perceived outdated topics | | ✓ | | | | ✓ | ✓ |
| | • Perceived broad content | | ✓ | | | | | |
| (ii) **People** | • Unfamiliar with the program and its rationales | ✓ | ✓ | | | | | |
| | • Lack of training | ✓ | | ✓ | | | ✓ | |
| | • Task oriented | ✓ | | | | | | |
| | • Lack of appreciation | ✓ | | | | | | |
| | • Unfamiliar with all students in class | | ✓ | | | | | |
| | • Some students are less motivated and involved | ✓ | ✓ | ✓ | | ✓ | ✓ | |
| | • Some students are overactive during lesson | | ✓ | | | ✓ | | |
| | • Unable to maintain class discipline | | | ✓ | | ✓ | | |
| | • Lack of teaching skills | | | ✓ | | | | |
| | • Some students have behavioral problems | | | ✓ | | ✓ | | ✓ |
| | • Students refuse to complete assigned tasks | | | | | ✓ | | |
| | • Students have negative opinions | | | | | ✓ | | |
| | • Teachers not suitable to teach program | | | | ✓ | | | |
| (iii) **Process** | • Insufficient time to run the program | ✓ | ✓ | | ✓ | | ✓ | |
| | • No periodic updating of the teaching materials | ✓ | ✓ | | | | | |
| | • No long-term follow-up for students | ✓ | | | | | | |
| | • Additional coordination work | | | | | | ✓ | |
| | • Involvement of several parties in school | | | | | | ✓ | |
| (iv) **Policy** | • No objective assessment on program evaluation | ✓ | ✓ | ✓ | | | | |
| | • No regular team meeting | | ✓ | | | | | |
| | • Lack of documentation of administrative procedure | | ✓ | | | | | |
| | • Large class size | | | ✓ | ✓ | | | |
| (v) **Place** | • Insufficient hardware equipment for running activities | | ✓ | | | | | ✓ |

**TABLE 4**
**Policy Support and People Factors in Each School**

| School | Policy Support and People Factors |
|---|---|
| A | • **Policy support**: The program was integrated into existing school lessons with clear manpower deployment. Involvement of class teachers and specialist teachers. A team was set up to coordinate the program. There were periodic meetings for evaluation and documentation of administrative procedure.<br>• **People**: Strong support from principal and coordinator. All teachers were passionate and motivated to deliver the program. |
| B | • **Policy support**: The program was integrated into existing school subject with clear manpower deployment. Involvement of counseling teachers. There was a preparation meeting before program implementation.<br>• **People**: Strong support from principal and coordinator. All teachers supported the rationale of the program and had a mission to deliver the program. |
| C | • **Policy support**: The program was integrated into existing school subject with clear manpower deployment. Deployment of teaching assistants to assist instructors. Provision of space and time for teachers to attend preparation meetings before program implementation, and periodic evaluation meetings.<br>• **People**: Vice-principal strongly supported the adoption of the program. All teachers supported the rationale of the program and were dedicated to deliver the program. |
| D | • **Policy support**: The program was integrated into existing school subject with shared workload among colleagues. Involvement of teachers who had vision and passion for youth development. Prior planning and providing sufficient time for teachers to do preparation.<br>• **People**: Administrative support from the principal and continuous support from coordinator. All teachers supported the program rationales. They were responsible, committed, and with mission and heart. |
| E | • **Policy support**: The program was integrated into existing school subject with apparent manpower deployment. Frequent informal meetings for preparation and evaluation.<br>• **People**: Both principal and coordinator supported the program rationales and took initiative to join the program. All the teachers who have heart and passion were selected to teach the program. They also had good cooperation. |
| F | • **Policy support**: The program was integrated into existing school subject with involvement of both class teachers and specialist teachers. Based on the experience of the Experimental Implementation Phase, the school strongly supported the implementation of the 20-h full program for the benefit of students. There were continuous quality improvement attempts.<br>• **People**: Strong support from the principal who took initiative to join the program and facilitated teachers' involvement and sense of ownership to the program. Strong support from the coordinator. All instructors agreed with the program rationales and were devoted to program delivery. |
| G | • **Policy support**: The program was integrated into existing school subject with a consistent pair of instructors responsible for teaching. Prior planning was carried out by principal and vice-principal. A room was provided for the social worker to handle related matters.<br>• **People**: Strong support from and involvement of principal and vice-principal. Both vice-principal and social worker had positive beliefs about students and were passionate to deliver the program. |

**TABLE 5**
**Perception of Program Integration**

| School | Perception of Program Integration |
|---|---|
| A | **Arrangement**: The program was arranged to fit into the existing school lessons with similar content and the lessons were distributed according to the topics of the subjects.<br>• **Advantages**: (a) students valued the learning on positive development; (b) there was no need for much extra work to be undertaken.<br>• **Disadvantage**: Nil. |
| B | **Arrangement**: The program was incorporated into existing school system.<br>• **Advantages**: (a) using existing time slots for teaching; (b) the program topics could merge with the existing and the previous school-based curriculum; (c) ensured the teaching quality of the subject; (d) had a smooth implementation and good arrangement that did not disturb the school system and affect others.<br>• **Disadvantages**: (a) the previous tailor-made teaching materials made by the team could not be totally used; (b) the subject teachers felt that it was difficult to be familiar with all of the students; (c) the content was too broad and sometimes vague. |
| C | **Arrangement**: The program was incorporated into existing school system.<br>• **Advantages**: (a) provided a chance to support student's personal growth; (b) had sufficient support in terms of research results, reference materials, teaching tips and audio-visual materials, suggestions for teaching and learning activities; (c) the 3-day training for teachers provided briefings on the curriculum structure and sufficient psychological preparation for teachers.<br>• **Disadvantage**: teachers who were not familiar with interactive teaching methods suggested more training be provided. |
| D | **Arrangement**: The program was incorporated into the existing school lessons with similar content and topics.<br>• **Advantages**: (a) students valued the learning as the project was part of the normal curriculum; (b) avoided adding extra workload to teachers.<br>• **Disadvantage**: Nil. |
| E | **Arrangement**: The program was incorporated into the existing curriculum.<br>• **Advantages**: (a) did not demand that teachers arrange extra time to teach and they were more receptive to the program; (b) better use of resources and enhancing morale; (c) encouraged students' involvement and commitment to the program.<br>• **Disadvantage**: Nil. |
| F | **Arrangement**: Formally incorporated some of the program into the curriculum of Life Education.<br>• **Advantages**: (a) the program contents met the needs of the students; (b) the program topics could merge with the existing and the school-based curriculum; (c) some teachers' workload was reduced; (d) administration of the program implementation was streamlined.<br>• **Disadvantages**: (a) structural changes in administration to implement the program effectively were needed; (b) some previous tailor-made teaching materials prepared by the teachers could not be used; (c) some subject teachers felt it difficult to be familiar with all their students due to time constraints; (d) additional coordination work was needed; (e) three different parties were involved to teach the program; (f) some contents had to be revised to match with students' special needs. |
| G | **Arrangement**: Formally incorporated the program into the curriculum of Moral Education.<br>• **Advantages**: (a) no labeling effects; (b) students' resistance was low; (c) increased instructors' seriousness in delivering the program.<br>• **Disadvantage**: Nil. |

**TABLE 6**
**Program Implementation in Schools with Students Having Different Academic Performance**

| School | Program Implementation |
|---|---|
| **High academic performance** | • Two schools incorporated the program into formal curriculum to help students' personal growth.<br>• According to education reform, the program was suitable to be included in Liberal Studies.<br>• Both schools revealed that the program had positive impacts on students and enhanced teacher-student relationships. |
| **Medium academic performance** | • One school incorporated the program into formal curriculum.<br>• One school incorporated the program into both formal curriculum and class teachers' period. As class teachers were better acquainted with the students, the program was better implemented.<br>• Although students had different learning motivation and their participation varied with topics, they had positive changes after the program. |
| **Low academic performance** | • One school incorporated the program into formal curriculum and the vice-principal taught the program. This helped the vice-principal to get acquainted with students and build up a close relationship.<br>• Two schools incorporated the program into both formal curriculum and class teachers' period. As class teachers were better acquainted with the students, the program was better implemented.<br>• All of these schools perceived students' growth and improvement in several aspects. Also, the instructors felt that they benefited from the program, and most of them were happy to have a closer relationship with students and better understanding of their students. The school atmosphere was found to be improved. |

**TABLE 7**
**Program Implementers' and Participants' Perception of the Program**

| School | Instructors' and Students' Responses |
|---|---|
| **A** | • Teachers had positive comments on the program, but they rarely expressed their appreciation.<br>• Students liked activities and novel topics. Their interest and involvement varied, depending on the topics.<br>• Some students were not used to be open in sharing themselves. Some students were not cooperative in class. |
| **B** | • Teachers had quite positive comments on the program.<br>• Students liked different activities. Their interest and involvement varied, depending on the topics.<br>• Students were interested in teachers' personal stories and sharing**.** |
| **C** | • The coordinator emphasized and praised students' participation in the class. |
| **D** | • Teachers had positive comments on the program. |
| **E** | • Teachers had very positive comments on the program.<br>• Students liked to attend the program and they were involved. They would apply what they had learned from the program. |
| **F** | • Teachers had positive comments on the program.<br>• Students' involvement varied:<br>1. All of them liked activities and novel topics.<br>2. Their learning motivation depended on the topics to be learned.<br>3. Some students did not feel comfortable in sharing about themselves in class and some were not cooperative. Some S1 students were considered as having inappropriate learning attitudes as they knew that their performance in P.A.T.H.S. program would not be assessed.<br>4. Some students considered that the program contents were just common sense. |
| **G** | • Teachers had very positive comments on the program.<br>• Students liked teachers' sharing and they were involved. They also enjoyed the activities. |

**TABLE 8**
**Perceived Program Effectiveness and Success**

| School | Perceived Program Effectiveness and Success |
|---|---|
| A | • The contact person had no clear answer on effectiveness, but perceived the program to be successful if students had improvement.<br>• Another teacher perceived it was successful, which was reflected from student involvement and program design.<br>• Some teachers perceived the program as beneficial to students; it was a good opportunity for students to come across this subject matter, for students' learning, and for teachers to get acquainted with students. |
| B | • The contact person had no clear answer on effectiveness, but perceived the program to be successful if students had improvement.<br>• Some teachers were unsure about the changes in students.<br>• Some teachers perceived it was successful and beneficial to students, which was reflected from student response and the school atmosphere. |
| C | • The program was well received by teachers. |
| D | • All teachers and social workers revealed that the program had positive impacts on students, e.g., improved emotional control and family relationships, more mutual understanding in class, sharing, respecting life, obeying rules, etc.<br>• Teachers perceived that the program had enhanced teacher-student relationships. |
| E | • Teachers perceived that the program was successful and the program effect was positive.<br>• Teachers perceived it was a happy learning experience for both teachers and students.<br>• Teacher-student relationships have improved.<br>• Students learned from each other, remembered what they had learned, had better emotional control, had high attendance rates and participation, were able to distinguish between right and wrong, etc.<br>• Teachers also witnessed changes and growth of themselves. |
| F | • The teachers perceived that the program was beneficial to students and it was a good opportunity for students to learn the subject matters and for teachers to get acquainted with students.<br>• Interviewees were unsure about the actual changes in students' behaviors. They thought that it was difficult to tell the effectiveness of the program at the moment, but the program was helpful to students because it provided an opportunity for students to reflect and knowledge gained might be helpful in the future.<br>• The contact person provided no clear answer on program success, but perceived the program to be successful as students had shown improvement.<br>• Interviewees quoted some examples of students keeping some teaching materials in their pockets.<br>• Social workers believed that the program was a portfolio of students' personal growth and beneficial to their growth. |
| G | • Both teacher and social worker perceived program effectiveness in terms of students' improvement and high involvement.<br>• Both teacher and social worker perceived program success in terms of students' improvement and parents' recognition. |
